# Curiosity and Creative Experimentation Among Psychiatrists in India

**DOI:** 10.1007/s11013-023-09829-1

**Published:** 2023-09-15

**Authors:** Claudia Lang, Murphy Halliburton

**Affiliations:** 1 Institute of Anthropology, University of Leipzig, Leipzig, Germany; 2 Department of Anthropology, Queens College and the Graduate Center, City University of New York, New York, USA

**Keywords:** Psychiatry, India, Ayurveda, Ritual healing, Creativity

## Abstract

Medical anthropologists have not paid enough attention to the variation at the level of the individual practitioners of biomedicine, and anthropological critiques of biomedical psychiatry as it is practiced in settings outside the Global North have tended to depict psychiatrists in monolithic terms. In this article, we attempt to demonstrate that, at least in the case of India, some psychiatrists perceive limitations in the biomedical model and the cultural assumptions behind biomedical practices and ideologies. This paper focuses on three practitioners who supplement their own practices with local and alternative healing modalities derived from South Asian psychologies, philosophies, systems of medicine and religious and ritual practices. The diverging psychiatric practices in this paper represent a rough continuum. They range from a bold and confident psychiatrist who uses various techniques including ritual healing to another who yearns to incorporate more Indian philosophy and psychology in psychiatric practice and encourages students of ayurvedic medicine to more fully embrace the science they are learning to a less proactive psychiatrist who does not describe a desire to change his practice but who is respectful and accepting of ayurvedic treatments that some patients also undergo. Rather than simply applying a hegemonic biomedical psychiatry, these psychiatrists offer the possibility of a more locally-attuned, context sensitive psychiatric practice.

## Introduction

Medical anthropologists know well that despite its official discourse as a singular, objective and universal approach to the body and pathology, biomedicine is in fact quite varied in its practices around the world (Jordan & Davis-Floyd, 1993; Lock & Nguyen, 2010; Street, 2014). Less attention has been paid, though, to the variation at the level of the individual practitioners of biomedicine. Anthropological critiques of biomedical psychiatry as it is practiced in settings outside the Global North have tended to depict psychiatrists in monolithic terms. The psychiatrist is rarely presented as an individual but rather is a modal and hegemonic technician of the mind who applies biomedical psychiatric protocols uncritically. This is not so much explicitly stated as implied in depictions of psychiatry and the lack of attention to individual psychiatrists and their own idiosyncracies and critical perspectives on their work (for a notable exception Pinto, 2019). This can especially be seen in medical anthropological analyses of psychiatry in India.

This article attempts to demonstrate that, at least in the case of India, some psychiatrists perceive limitations in the biomedical model and the cultural assumptions behind biomedical practices and ideologies. These psychiatrists supplement their own practices with local and alternative healing modalities derived from South Asian psychologies, philosophies, systems of medicine and religious and ritual practices. In our separate research projects on biomedical psychiatry, ayurvedic medicine and related topics in Kerala, India, we have each come to know psychiatrists who practice in mainstream psychiatric facilities but supplement their biomedical interventions with other healing modalities or, in some cases, speak of a yearning to diversify their practice in this way. These therapists seemed unsatisfied with how their work had been reduced to simple medication management, and expressed a desire to include more talk therapy and/or draw in South Asian disciplines of the mind from classical philosophy to Buddhism to ayurvedic medicine to contemporary ritual healing practices. The diverging psychiatric practices in this paper by three practitioners represent a rough continuum. They range from a bold and confident psychiatrist who uses various techniques including ritual healing to another who years to incorporate more Indian philosophy and psychology in psychiatric practice and encourages students of ayurvedic medicine to more fully embrace the science they are learning to a less proactive psychiatrist who does not describe a desire to change his practice but who is respectful and accepting of ayurvedic treatments that some patients also undergo.

## Psychiatrists in Medical Anthropology

In medical anthropology, psychiatrists often appear as interchangeable representatives of a hegemonic practice—recalling Durkheim’s (2014 [1893]) depiction of mechanical solidarity or “solidarity by similarities” (p. 57)—though on occasion they are considered as individual agents who reflect on or critically appraise the medical practice they have been trained in. Sometimes this takes the form of a critique of what they see as an overreliance on medication at the expense of other modalities psychiatrists are trained to use. We rarely see psychiatrists who are open to, curious about, or even engaging in nonbiomedical healing systems.

One of our earlier publications on mental healing in Kerala, India provides an example of this kind of portrayal. In Halliburton’s (2009) study of biomedical, ayurvedic and religious approaches to treating psychopathology, psychiatrists are depicted as technicians who conform to or explain psychiatric practice, as nodal points in a network of hegemonic practices. Aside from a psychiatrist who described how psychiatrists in India work more with family members compared to the US (where this psychiatrist also practiced), doctors/healers are presented as agentive, reflective individuals only in the depictions of ayurvedic medicine and ritual healing (Halliburton, 2009). We do not meet psychiatrists who critically appraise psychiatric practices or show an interest in other systems of healing.

In two related articles, Jain and Jadhav (2008, 2009) offer important assessments of psychiatric practice in India. They examine the approach to community psychiatry at sites in northern India where the “community” is simply the place where psychiatry is practiced, rather than a context whose sociocultural dynamics can impact mental health and healing. They also describe community psychiatry in Uttar Pradesh as “a top-down model conceived and written at the center, marginalizing the communities for which the mental health services are to be made available” (2008, p. 563) and suggest that “psychotropic medication has become the essence and embodiment of India’s community mental health policy” (2009, p. 61). As in Halliburton (2009), psychiatry is depicted here as a practice and set of policies. Psychiatrists are interviewed to understand the history of community psychiatry, but we do not get to know individual psychiatrists as reflective or critical agents. Psychiatrists appear to consent to and uncritically advocate these policies and practices. This may well be what they do in this instance, and certainly in our own work, we have seen that the administration of medication is “the essence and embodiment” of psychiatry in Kerala. Yet some psychiatrists we met in Kerala object to the narrow pharmacological approach to psychiatry and would likely welcome some of the critiques and suggestions offered by Jain and Jadhav. Some even go further in demonstrating curiosity and creativity in engaging with nonbiomedical therapeutic modalities.

Nunley’s “Why Psychiatrists in India Prescribe So Many Drugs” (1996) observed the same heavy reliance on medication as well as a frequent resort to the use of electroconvulsive therapy in Indian psychiatry, and attempted to explain these practices from a variety of angles. Interviews with individual psychiatrists are cited regularly in this article, but always to explain why they and their colleagues focus almost exclusively on medication. They explain that they are responding to patient expectations, that they tend to see more severe cases than elsewhere as milder cases are managed through other means, and they offer other rationales. None stray from applying and defending the biomedical paradigm, although Nunley does also report on a survey he conducted showing that psychiatrists felt that what he referred to as “faith healers” were “sometimes good psychotherapists” who “may benefit” patients with mild problems (185). This indicates a mild tolerance for certain other types of healers, similar to what we see with Dr. Achuthan below, although not the kind of endorsement or bold interest in applying alternatives of the other two psychiatrists we present. Nunley’s analysis appears to address only views about “faith healers” which we understand to be a term for a variety of religious or ritual healing methods, while the psychiatrists in this article address both a kind of ritual healing and Ayurveda toward which psychiatrists may have different attitudes.

Mills (2014) offers important challenges to the Movement for Global Mental Health and other actors who are promoting the expansion of biomedical psychiatry in the “majority world.” In her analysis, we meet a few NGO psychiatrists who do roughly what the psychiatrists mentioned in the literature above do: articulate the biomedical paradigm and lament that their NGO ended up overly focused on dispensing medication. We do also hear critical voices from within psychiatry taking on central assumptions and practices of this discipline, but these come from published critiques by psychiatrists, such as Breggin (1991) and Moncrieff (2008), who question the dominant biogenetic approach to understanding psychopathology, as well as Fanon (2008 [1952]) and Fernando (2019 [1991]), who critique the practice of psychiatry in the colonial and postcolonial contexts. Some of these critics are part of an anti-psychiatry movement within psychiatry that has a long pedigree (Robcis, 2021; Szasz, 1961).^1^ These critics from within the profession take aim at the overuse of medication and even the biomedical paradigm itself, but they do not indulge nonbiomedical healing approaches such as Ayurveda or ritual healing as some of the psychiatrists we met in Kerala have. And while psychiatrists as academic interlocutors are sometimes engaged in medical anthropology and related fields, it is the *psychiatrist as ethnographic subject* that is more rarely seen as a critic or a subject who is curious about alternatives.^2^

Varma (2020) describes psychiatric and psychotherapeutic care in a context of military occupation in Kashmir. In a situation where, as Varma argues, mental health care has also become a political technology of the colonizing power, doctors regularly apply electroconvulsive therapy (shock treatment) as a treatment modality. While many patients understand this technology as linked to military torture methods, psychiatrists saw ECT as a cheap, safe and effective form of care for severe mental illnesses. Here, too, psychiatrists are depicted as homogenous and as enthusiastically and uncritically supporting this contested technology, although we also encounter one psychiatrist Dr Abdul who worked for an international NGO with a more psychosocial approach, and who criticized biological psychiatry’s model of diagnosing and medicalizing by instead stressing nonjudgmental listening, empathy and the attention to local knowledge and needs.

We take inspiration from Pinto’s historical analysis *The Doctor and Mrs. A.* (2019). Drawing from a published psychoanalysis, Pinto explored the conversation between a young woman with a psychoanalytic psychiatrist as a window onto gender, sexuality and ethics in late colonial Indian society. Putting Mrs. A. and the psychiatrist Satya Nand at the center, this book not only shows desires and imaginations as deeply entangled with social, cultural and historical context. It is also an exceptional example of putting an individual psychiatrist’s innovation and creativity in experimenting with dream analysis at the center of anthropological interest.

Moving outside of India, we again see psychiatrists depicted as technicians of biomedicine rather than as individual, critical or creative subjects. In Biehl’s *Vita* (2013 [2005]), we are told the story of Catarina who is abandoned by her family and society to an asylum known as Vita. The psychiatrists who treat—or rather, manage—Catarina are referred to and quoted, usually through the notations made in her chart or comments made in conversation with Biehl about Catarina. Individual psychiatrists are named, but they are depicted as people who simply apply the biomedical and neoliberalizing healthcare system in Brazil that Biehl critiques. They comment upon the status of patients, explain why patients were admitted, and renew prescriptions. Two individuals we do get to know in the discussion of psychiatric care who are critical of mainstream practices are, significantly, psychologists, one who runs a mental health reform movement and another who directed an alternative psychosocial rehabilitation center (pp. 123–178).

In his analysis of the dysfunctional patchwork of services that constitutes community psychiatry in the United States, Brodwin (2013) focuses on the experience of frontline mental health workers and the patient/clients whose lives they struggle to manage. Psychiatry is described in general terms, and psychiatrists are somewhat removed from a narrative that focuses on the experience of case workers who interact directly with clients. We do though get extensive exposure to a psychiatrist Dr. Young, who appears as a vehement advocate of the biomedical model interpreting all behavior reported by case workers in terms of medication management issues. He is the paradigmatic adherent who endorses, enforces and teaches the biomedical model.

The depictions of psychiatrists in these works does not necessarily amount to an oversight, an overlooking of dissenting voices. What we see in the works depicted above may be exactly how things are in these settings, and they may well represent the subjectivity of most psychiatrists in those contexts. In much of this work as well, there is a tendency, as there is arguably in medical anthropology in general, to focus on the world of the patient more than the world of the psychiatrist when researchers consider the topics of mental health and illness. This may lead us to focus more on the diversity of experience among patients than among doctors. While our work has focused both on the experience of patients and the practices of healers (Halliburton, 2009; Lang, 2018), we each started to notice that some psychiatrists showed us they could be more complex in their work as healers than mere applicators of biomedicine. They could be subjects with diverse interests, curiosities and creativities. This may be partly because we had both written on ayurvedic medicine and ritual healing, which may have led some psychiatrists to be more forthcoming with us about their interest in Ayurveda and other healing modalities.

The context of Kerala, and India more broadly, may help explain why these observations about psychiatrists’ interests in modalities outside biomedicine comes from fieldwork in India, rather than Brazil or the US. In India, there are a variety of local alternative approaches to healing to draw from including an institutionalized local medical system that has significant legitimacy in the form of ayurvedic medicine which has its own colleges and hospitals and is recognized by the Indian government as a *bona fide* Indian system of medicine, meaning that it obtains some government funding and support. Ayurveda offers an alternative approach to psychopathology that, like biomedicine, is based on a pharmacology and a theory of physiology and mental processes. Additionally, local philosophies of the mind, such as Advaita Vedanta which is invoked by two of the psychiatrists presented below, offer a discourse that is not biomedical but also not marginal. Advaita Vedanta is well known and prestigious as a way of understanding the self and suffering. Alternative discourses and paradigms such as these may be more marginalized or in some ways harder to draw upon in the biomedical sector in other places.

China offers a context similar to that of India with the practice of traditional Chinese medicine^3^ as a mainstream complementary, or sometimes rival, medicine to biomedicine. Research on health and illness in China has described relations between biomedicine and Chinese medicine from a variety of angles. Zhan (2009) depicted practitioners of Chinese medicine who adopt practices from biomedicine and other kinds of hybridization in clinical practice in China including hospitals that offer both biomedicine and traditional Chinese medicine. The kind of creativity and medical syncretism portrayed in this work is the doctor of Chinese medicine who brings in biomedical techniques, the reverse of the kind of experimentation we have observed, and Zhan’s work focuses on general medical practitioners, not psychiatry. Some scholarship has examined differences between biomedical and traditional Chinese medical approaches to mental health, but these outline a general debate (Baum, 2022) or attempt to explain the areas of overlap and distinction between concepts of the mind and pathology in the two systems (Scheid, 2013). We have also seen how people suffering mental distress in China reconcile the individual self of western psychology with Chinese expectations about the self’s relation to the social (Zhang, 2020). Lee (1999) does present individual, agential psychiatrists in China but only in terms of their views on the commercialization of psychiatry and psychiatrists’ relations to the pharmaceutical industry. We do not see biomedical psychiatrists exhibiting creativity and curiosity and improvising with Chinese medicine or other local healing modalities for mental health. Such practitioners may be well represented in China though they have not yet been brought to the fore in medical anthropological literature.^4^

Jain and Jadhav’s critiques of psychiatry in India offer suggestions for improvement including “that consideration be given to … alternative models” (2008, p. 579) and to “multiple models for mental health services (2009, p. 77). As examples of such models, they cite innovative mental health programs in India, such as Banyan in Chennai and the Bapu Trust in Pune, that offer diverse and empowering interventions. Psychiatrists we spoke to in Kerala who are presented here and who work in institutions that have entrenched the same practices Jain and Jadhav critique, may welcome such suggestions. They show interest in multiple modalities of mental health care even if they are often constrained by the dominance of medication management in their jobs. Although the cases we present here are located in Kerala, some rare cases of curiosity and experimentation amongst psychiatrists have also been described in other contexts. For example, in their work on the “walking corpse syndrome”, a complex of cognitive difficulties, impulsive behavior and dissociation amongst Sri Lankan Tamils, Affleck et al. (2022) show how Tamil psychiatrists include meditation and yoga in their treatment, next to pharmacological treatment and cognitive behavioral therapy. Similarly, Bangalore’s biomedically-oriented National Institute of Mental Health and Neurosciences integrates Yoga practices to a certain extent.

## The Conditions of Psychiatric Labor

It is not surprising that some psychiatrists might feel unsatisfied with their work given the assembly line style practice of medication management we and other researchers (Jain & Jadhav, 2009; Nunley, 1996; Pinto, 2014, Varma, 2020) have observed in Indian psychiatry. In 2014 and 2016 in southern Kerala, we accompanied District Mental Health Program (DMHP) staff on their visits to community health centers to observe some of the occasional psychiatric outpatient clinics they held there. In a visit to a neighborhood clinic, Murphy observed a psychiatrist interact with 39 patients in just under two hours. Each session lasted 1 to 3 minutes and the longest was 5 minutes. The psychiatrist usually asked a few brief questions about the patient’s general state, about their sleep and side effects of drugs they are taking. He would then write or renew a prescription and pass the patient’s case book to a social work student who entered details about the consultation in a register. Sometimes the psychiatrist would review additional documents, such as a CT scan. To one patient, he gave an activity sheet to record activities, mainly related to getting a job. Aside from this one gesture at a social approach to treatment, all of the cases, which included diagnoses of anxiety, depression, schizophrenia, bipolar disorder and epilepsy, focused exclusively on medication. This had slightly changed in 2017 when Claudia accompanied the same team that now included a clinical psychologist and a social worker. Sometimes, patients were examined by one of them first for a detailed assessment before their file was handed over to the psychiatrist who wrote the diagnosis and prescribed the medication under similar time constraints that we described above. Adults’ follow up visits were mostly to the psychiatrist. The psychologist’s involvement was mostly limited to the few children who showed up in the clinic. She spent considerably more time with them, and treatment was mostly focused on behavioral modification and only rarely included medication. Another change has come with the *Ashwasam* program in 2018 that introduced the treatment of depression into general primary health care in Primary Health Centers. As part of this program, staff nurses are supposed to provide counseling, though this is often dropped in busy health centers’ practice. However, when we look at the grassroot level of primary health care in Kerala—community health workers, i.e. outreach workers with more basic training than the mental health staff described above—we find a less medicalized and more social approach to mental health (Lang, 2019).

Intake sessions were not much different at the government and private mental hospitals where we conducted fieldwork, except in those settings they usually lasted a few minutes longer, especially during the first intake. Many psychiatrists and psychologists who work for government facilities also hold private therapy sessions. They do so usually from home offices where they can take more time to engage in talk therapy although queues at these private clinics can also be long and the sessions shorter than what some therapists said would be the ideal.

Several psychiatrists who worked in these conditions complained to us about how they could not use some of the methods they had trained for, most notably psychotherapy, because of the volume of patients they had to see. Others experimented with unconventional methods they had made themselves familiar with in the course of their career. It was clear that the emphasis on medication was not only due to the ideology of the therapists involved in outpatient public clinical practice or patients’ expectations, but also to the length of the queues they had to contend with. The psychiatrist from the DMHP OP session explained in an interview, that he considers psychological and social factors important to understanding and treating mental illness and even pointed out the activity sheets they use to understand the patients’ behavior and social and work life which he utilized only once during this OP session described above. In addition, some psychiatrists were aware of the rich heritage in healing systems that exists in India—from Ayurveda to Siddha medicine to psycho-philosophical disciplines such as Advaita Vedanta to Buddhist orientations to mind and self to Christian charismatic healing. Some had respect for and interest in such practices, in particular ayurvedic medicine’s techniques for treating mental problems. This paper presents examples of psychiatrists who work in mainstream biomedical institutions, but who are interested in, yearned for, or actually utilized and experimented with nonbiomedical therapeutic recourses for mental problems. Their practices reflect medical anthropological critiques that question a reductionist biomedical model and the growing hegemony of psychiatric practices while pointing to more holistic orientations and the virtues of medical pluralism. A focus on individual doctors’ actual clinical practice of experimentally or theoretically engaging with spirits, ritual healing, hypnotherapy or Ayurveda as non-institutionalized practices within institutional psychiatry troubles not only homogenizing narratives of psychiatry in India but also a clear-cut boundary between psychiatry and alternative healing approaches. The three case studies emerged out of our individual research on mental health in Kerala. Each of us has done participant observation and interviews in biopsychiatric and Ayurveda mental hospitals, in community mental health care, and with various ritual healing practitioners. The discussion with Dr. John is based on participant observation with Rajini’s treatment and several audio-recorded conversations between Dr. John and Claudia. In these conversations, Dr John explained how he struggled to make sense of what was happening with Rajini and how to best help her. They also turned more generally around the spiritual dimension of mental illness, religion and spirit possession. On several occasions, he also explicitly asked Claudia for an opinion which brought her to the limits of medical anthropological knowledge and its possible application. She also observed Dr John interacting with other patients and discussed cases with him. Although in general, he was keen to take more time for his patients and include psychotherapy as much as possible within the constraints of the busy hospital routine, his treatment of Rajini was exceptional. The discussion with Dr. Achutan was based on a semi-structured interview by Murphy, that was audio-recorded, and several informal conversations with Dr. Achutan at the mental hospital where he works before and after the formal interview. The discussion of Dr. Menon is based on multiple conversations that were not recorded other than through hand written fieldnotes. Observations were also derived from “participant observation” which involved a variety of scenarios where Murphy would spend time at the hospital observing what was happening in addition to conducting interviews. On several such occasions, Dr. Menon invited Murphy to converse about mental health, conversations which often turned to Dr. Menon’s interests in classical Indian philosophy, such as Nyaya, Vaisesika or Advaita Vedanta. As another example of the fortuitous encounters involved in participant observation, on one visit to the hospital by Murphy, students from the local Ayurveda college were also visiting, and Dr. Menon introduced him and the students to each other, which provoked a conversation between Dr. Menon, Murphy and the students regarding ayurvedic and biomedical approaches to treating psychopathology.

### Dr. John

The following case represents the most bold and creative departure from standard psychiatric practice. It depicts the entry of religious healing into the spaces of institutionalized psychiatry and with a psychiatrist’s ways of experimenting as he navigated epistemological, ontological and clinical possibilities and uncertainties. Dr. John was a senior psychiatrist at a public mental hospital in Kerala when Claudia met him in October 2016. She used to sit with him when he saw inpatients and outpatients often discussing cases together. His busy schedule did not allow him to spend as much time for single patients as he would have liked to but when patients came back for follow-ups, often in the company of their family, he spent considerable time with them. One of the patients Claudia followed closely was Rajini. Rajini was an exceptional case. She accommodated several spirits and deities that regularly spoke through her in multiple ways. Claudia followed Dr John’s treatment of Rajini at the mental hospital over several weeks and attended the psychiatrists’ discussions and their struggles for Rajini’s diagnosis and cure. In spite of larger reforms in the last 15 years or so, the large mental hospital where Dr. John was working was still characterized by a heavy reliance on pharmacotherapy and electro convulsive therapy (ECT)—in spite of the presence of some few psychiatric social workers and clinical psychologists. In the absence of psychotherapy, the social workers focused on administrative issues and the clinical psychologists were mostly concerned with intelligence testing.^5^

Rajini, a woman in her forties, and her husband Raman were from lower-socio economic background and hailed from a neighboring state. In eliciting Rajini’s history, Dr John learnt that she had worked as an assistant in a school for differently abled children but was forced to quit due to frequent possession attacks that increasingly disrupted her work. The visible manifestation of gods and goddesses in a human body is part of Hindu religious experience and Rajini told Dr John that her mother used to get regularly possessed during religious festivals. Rajini had gained some reputation and money when she started to get possessed by the goddess Bhadrakali who spoke through her to those who came to seek her help. Problems, the couple told Dr John, started only when other spirits and deities—such as *nagas* (snake deities) and ancestor spirits of former members of the royal family of Travancore—started using Rajini’s body and voice to express their desires. Dr John observed Rajini frequently “switching” into these deities and spirits. He and the two other psychiatrists involved in Rajini’s treatment considered these possessions as “defense mechanisms” that protected her from falling into depression and committing suicide. Yet, Dr. John also considered the possibility of a “real possession”.

Dr. John had an interest in the spiritual dimension of mental illness and in the topics of religion, spirit possession and mental illness in Kerala. Rajini was an interesting patient for him and he was curious to experiment with different therapeutic approaches. Like other psychiatrists in India, he had not been trained, he complained, to deal with religious matters in clinical practice. This lacuna is a problem, he and several other psychiatrists complained, especially in India, where religious and spiritual practice are part of the everyday for most people, and largely frame how people know, experience, express and get cured from mental health problems. As one Indian psychiatrist at another private hospital in Kerala expressed it,

While to patients with Western education, I can frame my treatment and counseling as self-actualization, as a way to expand their potential; to a patient who is not exposed to Western culture, I will frame it in terms of achieving peace or harmony inside and getting closer to God. So we have to bring God and religion and religious texts into our treatment.

The way that Dr. John approached and treated Rajini was exceptional for psychiatry in India where, as we indicated above, psychiatric encounters are often limited to a few minutes per patient and treatment heavily relies on pharmaceuticals and sometimes ECT with little time and motivation for counseling or psychotherapy. A patient complaining of spirit possession is exceptional in psychiatric institutions in India. While possession is a common phenomenon outside psychiatric spaces in religious healing centers, medical anthropologists have argued that patients know what different institutions “ask for” and usually do not get possessed in psychiatric institutions (Naraindas et al., 2014). Rajini’s case was unusual, first for the happening of spirit possession within the spaces of institutionalized psychiatry, and second for a psychiatrist who navigated and tinkered with various diagnoses and treatments at the intersection of psychiatry, psychotherapy and religion.

Dr. John started Rajini’s inpatient treatment with what he called “relaxation therapy”, a merger of Jacobson relaxation technique, guided imaginary and hypnosis. Since this was not part of the usual treatment line and therefore the hospital had no spatial provisions, these treatments somehow ironically took place in the “shock room” in which many other patients got electro convulsive therapy twice a week. The relaxation therapy was a way for the psychiatrist to learn more about Rajini’s “subconscious conflicts” (that he elicited from her through questioning her in a state of relaxation). At the same time, the therapy’s purpose was to empower Rajini psychologically and spiritually by increasing her inner strength and “recharging” her through the control of the deities and spirits in her. Using the metaphor of electric current, he explained to Rajini the effects of his “recharging”, while she was lying relaxed:

What do we do when the power of a battery is exhausted? We connect it to an electrical power source. After some time, the battery gets charged. Like that you will get the charge through me. What is this charge for? It is to give you the power to prevent the goddess and other forces coming in to you.

Dr. John also tried to educate Rajini in basic biodynamic mechanisms of externalizing her inner conflicts into external entities, by telling her that the spirits and deities were in fact psychological defense mechanisms. Unbearable memories, emotions and experiences of abuse, he told Rajini, made her “switch into alters”. He tried to convince her that by “switching” into powerful spirits, she gained agency and power where in her ordinary state she was powerless and helpless. Agency, for example, against her dominant and sometimes abusive twenty years older husband who used to respect and obey her only when the goddess Bhadrakali or the other spirits appeared in his wife. Agency also to contradict the junior male doctor when he was trying to probe into her marital and sexual history and Bhadrakali told him (through Rajini) that these were inappropriate questions.

Rajini’s “switching” into powerful agencies whenever emotions became too painful also complicated Dr. John’s efforts of hypnotherapy, as it was difficult for her to reach a deeply relaxed state as a prerequisite for the hypnosis to be effective. As a result, Dr. John resorted a few times to a next step and assumed the role of an exorcist similar to what we have seen in the context of other Christian practices of deliverance and exorcism in Kerala. As a practicing Christian, Dr John used to attend charismatic healing sessions in his personal life and has seen exorcisms and deliverance prayers. Sitting opposite to Rajini before he began the first session, he explained,

It is the real God who made me a doctor for the purpose of saving people like you. That God is telling you this through me: ‘Rajini, you need not obey to other forces. I will talk to you now through this doctor. You will get strong if you listen to this doctor.’ I have been made a doctor by God. Don’t change into *amma* [Bhadrakali], *thampuratti* [princess] or *naga* [snake] during this time [alluding to the spiritual entities into which she used to ‘switch’].

Dr. John was a Christian and in his encounters with Rajini, a Hindu, he merged his medical authority with borrowed authority from the Christian god. His psychological treatment became a performance of spiritual supremacy of the Christian god over Hindu deities and ancestor spirits. Who and what was the object of Dr. John’s interventions? How did he understand his own practice? On the one hand, for the doctor, engaging Rajini’s own framing of spirit possession was primarily a psychological technique.

I want to tell her that I have the capacity to cast out these spirits. This is just a technique. I am using her own language. If I was just a simple doctor, I couldn’t do that. I have injected in her the idea that I am spiritually powerful and I can cast out these spirits. If we can successfully cast the spirits out, then our treatment will be a greater thing compared to what the *potties* [Brahmins who practice ritual healing] and religious people tried.

But his assumption of the role of an exorcist was also a spiritual technique since Dr. John himself did not completely discard the possibility of a real presence of spirits in Rajini. What was at stake in Rajini’s case, then, was never finally determined, ontological ambiguities were kept open. Spiritual, biodynamic and chemical processes were never rigorously kept apart, but were porous and ambiguous. And so was Dr. John’s treatment. If ontologies are enacted through therapeutic practice, then Dr. John’s various therapeutic practices not only created the same entities and processes in which they aimed to intervene. They also left ontological questions open by potentially bringing into existence spiritual, biodynamic and chemical effects.

After struggling for a diagnosis with the two junior psychiatrists for weeks and after ruling out schizophrenia and dissociative disorder, Dr. John finally reached a diagnosis: depression, although this also was preliminary. In a way, Rajini’s diagnosis never left the state of preliminarity. Dr John and his team struggled with the inadequacies of psychiatric diagnostic categories and pharmacotherapy in the context of spirit possession. They also struggled with the lack of insight of Rajini and her family into the need to continue and comply with the psychiatric and psychological treatment. And Claudia and Dr John struggled with the question of how to translate medical anthropological and cross-cultural psychiatric knowledge into actual clinical practice.

The psychiatrists prescribed Rajini two pharmaceuticals: an antidepressant and a benzodiazepine. The crux was that the suicide risk was high for Bhagavati and could even increase, Dr. John worried, should the therapeutic efforts of the relaxation be successful and deprive Rajini of the option to switch into powerful alters. Rajini’s doctors who were well-informed about medical anthropological and cross-cultural psychiatry’s critique of universalized psychiatric practice and in Rajini’s case sought to offer what they regarded as a “cultural sensitive” treatment, discussed possible ways for Rajini to keep her positive possessions while helping her to get rid of the negative ones. This was no easy task for several reasons, not least because it was difficult to discern what was positive and what was negative for Rajini in the first place. Apart from psychological, pharmacological, and spiritual treatment, Dr. John also addressed the social dimension of Rajini’s problem: he enrolled her at the weaving unit of the hospital and increased her meagre salary there out of his own pocket. Although his time resources were limited, he further planned to put her into contact with government welfare and poverty eradication schemes.

In the last session to which the couple had traveled from a neighboring state, Dr. John started with a breathing exercise. Drawing from Advaita Vedanta, Rajini and Raman were supposed to learn not to identify with their body and with their mind. Rajini should further learn to what Dr. John called “lock” and control the deities and spirits whenever they wanted to manifest within her with an imaginary key. By practicing this, the doctor assured her, she would gain power (*shakti*). Then he addressed Raman and advised him to show more love and respect to his wife in the future. His idea was that Rajini unconsciously used the powerful entities to get the affection and respect of her husband that he did not give her otherwise. Dr. John also told Rajini that instead of hoping for the property share that the ancestor princess’s spirit in her was aspiring for, she should find herself realistic ways to earn money and that he would try to help her with that. Then Dr. John asked the couple to reenact their marriage ceremony. The session culminated in a part when Rajini and Raman, while holding hands, told each other what they wanted from each other and what hurts them, and were asked to forgive each other in a way that we have seen in Christian Charismatic deliverance sessions, and finally were asked to hug and kiss. It was a session that creatively, experimentally and eclectically mixed mindfulness, couple therapy and Christian religious therapy.

Rajini’s treatment was a doctor’s effort to navigate through a complex case in which depression, psychiatry, gods and spirits, state bureaucracy, cross-cultural psychiatry and ontological pluralism were interwoven into a complex assemblage of multiple forms of care. The doctors’ therapeutic practices not only enacted multiple entities (psychological defense mechanisms, spirits and gods, physiological processes in the brain/neurochemical imbalance in the brain). They also navigated, stabilized, destroyed and rebuilt complex intersecting worlds.

Dr John’s treatment of Rajini was exceptional. Although he used to spend more time with his patients than other psychiatrists and he tried to include talk therapy techniques at least to a certain extent, his treatment did not usually include exorcism and Christian charismatic elements. The unique case of Rajini and her particular ailment is an example of the limits of biopsychiatry and Dr John’s efforts a case of creative practice negotiating its dead ends. This case of spirit possession in the spaces of a psychiatric hospital troubles homogenizing narratives of Indian psychiatry. Using the case of Dr. John’s treatment of Rajini and her treatment in a mental hospital, we have shown the epistemological, ontological and therapeutic struggles and ambiguities that may emerge when a psychiatrist tries to overcome the limitations of reductionist psychiatric practice and experiment with alternative methods.

### Dr. Menon

Dr. Menon also practiced psychiatry in a biomedical hospital. He did not consider exorcistic interventions along the lines that Dr. John employed, but he would agree with Dr. John’s invoking of Advaita Vedanta with Rajini and Raman as he felt such intellectual traditions of classic Indian philosophy could have a therapeutic effect on psychopathology. He was also not only respectful of but even enthusiastic about the capacity of ayurvedic medicine for addressing mental health problems. Ayurveda is a widely practiced indigenous medical system of South Asia which includes a pharmacopoeia along with other interventions in physiology, diet and lifestyle. It is taught and practiced in colleges and clinics and includes treatments for psychopathology for which the state of Kerala is widely reputed.^6^ A fan of classical Indian philosophers whose work informs Ayurveda, Dr. Menon would try to compel enthusiasm on the part of others about Ayurveda and the potential psychotherapeutic benefits of Indian philosophies. These included ayurvedic medical students who would visit his hospital to become familiar with allopathic approaches to treating psychopathology as part of their training at a local Ayurveda college.

On Murphy’s first meeting with Dr. Menon in 2014 at the hospital where he worked, they discussed classical Indian philosophy and Ayurveda and how philosophical systems like Nyaya and Samkhya—that contemplate the nature of matter, knowledge, perception, and the self—influenced the epistemology of ayurvedic medicine. Murphy explained how he thought the discourse on the nature of the mind, body, consciousness and emotions in the Upanisads, the classical texts representing the end of Vedic literature (circa 600 BCE) that continue to be widely read, can be therapeutic and seem to continue to resonate with contemporary audiences. He expressed his surprise that such disciplines of psychology were rarely employed by mental health professionals in India who tended to follow current protocols from the West, primarily the pharmaceutically-oriented biomedical model and, in the rare moments allotted to talk therapy usually in private practice sessions, the psychology of people like Carl Rogers or Marsha Linehan. Dr. Menon concurred and lamented this practice. When Dr. Menon invited Murphy to visit the outpatient psychiatric session at the hospital the same day, it was clear why it would be difficult to indulge in talk therapy of any kind.

Making his way through the crowd waiting to be seen by a psychiatrist (or families waiting to “show their ill relatives to the doctor” to use the phrasing in Malayalam), Murphy entered the hospital’s OP consulting room invited by Dr. Menon and joined him, other therapists, patients and family members around a large table where consultations occurred. Everyone chatted briefly mindful of the large crowd waiting for their consultations. As with the visit to a community clinic reported above, the psychiatrists were able to spend very little time with each patient, diagnosing or getting an update on the condition of the patient often by talking to family members, and prescribing, renewing or changing medication.

On subsequent visits to the hospital, Murphy would sometimes see Dr. Menon accompanied by or, on one occasion, surrounded at a desk by an entourage of students of ayurvedic medicine doing their biomedical psychiatry rotations as part of their training. When he saw Murphy on such occasions, he would hurriedly invite him over knowing that he could be an ally in convincing these students of the merits of Ayurveda for treating mental illness, and Dr. Menon would introduce Murphy as a foreign scholar who has researched and published on ayurvedic treatment for psychopathology. There is an impression in India that students who attend ayurvedic medical colleges were unable to gain admission to biomedical medical schools. While this appears to be true of most entrants to Bachelors of Ayurvedic Medical Science (BAMS) programs, there are also those who enter training in Ayurveda due to a commitment or calling to this discipline (Naraindas, 2006). The ayurvedic students who visited Dr. Menon’s facility exhibited a deference to biomedical views of psychopathology, which Dr. Menon pushed back against. This was not because Dr. Menon necessarily had any doubts about the biomedical perspective, but he felt Ayurveda also had a lot to offer as a therapeutic modality. He was even aware and would point out that the first antipsychotic in biomedical psychiatry, reserpine, was derived from an ayurvedic treatment for mental illness that utilizes as plant known as serpagandhi (rauwolfia serpentina) giving a biomedical scientific imprint of validity to a particular ayurvedic therapy.

Dr. Menon did not act on these beliefs by engaging or experimenting with Ayurveda or other modalities in his practice. He didn’t have time in his workday, and he may have been wary of straying from the boundaries of biomedical practice as a psychiatrist at a government run hospital. However, one could describe his regular encouragement and education about Ayurveda for ayurvedic students he advised as a way of acting on his endorsement of ayurvedic treatment. Dr. Menon did also speak of his interest in one day opening his own private clinic for treating mental illness, which would combine aspects of his biomedical training along with Ayurveda and insights from the psychological and philosophical traditions of India. Dr. Menon had not opened his own clinic by the time Murphy visited Kerala again five years after originally meeting him and hearing of these interests, but it remains an aspiration as he continues to work at the same biomedical institution. Dr. Menon’s interests come across as a yearning for something more than the routine he was involved in and required to follow at work, where a one-size-fits-all model of biomedical psychiatry that is almost completely about medication management is the expectation, and the patient queues remain long leaving little time for innovation and variation.

### Dr. Achutan

Dr. Achutan was a psychiatrist at a mental hospital in Kerala who would also occasionally see patients who were residing at a separate care and rehabilitation center. He praised the center for the quality of their care and how, in his opinion, the staff treated the residents like family. At this latter facility, residents were seen by doctors of ayurvedic medicine for their regular health needs since government regulations for such facilities allowed the use of biomedicine, also known as allopathy in India, and any of the bona fide Indian systems of medicine including Ayurveda to be used for the regular health care of their residents. The ayurvedic doctors also treated patients for side effects of the biomedical psychopharmaceuticals they were taking claiming that their treatment countered some of the fatigue and lethargy produced by allopathic drugs. Dr. Achuthan expressed his appreciation and respect for ayurvedic medicine but also conveyed his concern that he did not know what ayurvedic drugs were being used and whether they might have problematic interactions with allopathic drugs he was prescribing to the same patients. But after a while, as if concerned that he might be perceived as dismissive of Ayurveda, he affirmed:

Personally, I believe in all systems of medicine. I have a strong belief in Ayurveda. I believe in Siddha, especially Siddha medicine [a medical system practiced in southern India]. Especially in Siddha, they have topical ointments which...topical balms and ointments which are very effective. Massaging systems. That I believe, but from the information I get from literature, you ask me if you can mix, I think I may not [ie, he would not mix ayurvedic or Siddha drugs with allopathic medications].

Dr. Achuthan also praised the engagement with pets at the rehabilitation facility as a kind of therapy: “They have these pets with whom they can, over a period of time, develop some kind of a bond [...] [T]he pets might help the patients in creating some kind of emotional sharing, or soothing them or maybe making them more comfortable.” But foremost in his view of what these residents needed was to stick to the allopathic drug regimen, to continue taking the right medicines at the right times. Dr. Achuthan was a believer in psychiatry and did not evince interest in indulging in other treatment modalities in his own work, but he was clear to point out that he respected and even “believed in” Ayurveda and Siddha medicine. He also allowed that the empathy of the staff and the occupational and pet therapy were good for these residents, but he might have been skeptical of the religious healing modalities Dr. John engaged in. He was critical of religious framings of mental illness among family members of patients which he felt accounted for much of their resistance to the biomedical model. He observed “They still believe [...] it [mental illness] is either due to past sins or somebody has done something to the family [a reference to evil eye or sending sickness through sorcery].” Dr. Achuthan believes in, or allows for, the possible effectiveness of certain nonbiomedical modalities, but there is a limit to the range of what he considers credible in the end sticking to the standard protocols of allopathic treatment and not indicating a desire to use or experiment in interventions outside of that practice himself.

## Conclusion

While anthropological scholarship on psychiatric practice tends to depict patients as individuals, psychiatrists often appear as monolithic characters. In this paper, we have trained our ethnographic attention to individual psychiatrists’ approaches and practices. Attending to psychiatrists as individual actors rather than as modal technicians reveals psychiatric practice in India as less homogenous but more diverse, experimental and creative. This paper has focused on three psychiatrists whose individual approaches and practices do not conform with homogenized imaginaries of biopsychiatric practice in India. These psychiatrists perceive limitations in the biomedical model and the cultural assumptions behind biomedical practices and ideologies and supplement their own practices with local and alternative healing modalities derived from South Asian psychologies, philosophies, systems of medicine and religious and ritual practices. They creatively experimented with or at least were curious and sympathetic to South Asian approaches to mental health.

Looking at these individual practitioners, their practices and critique reveals interesting alignments with the critique of psychiatric practice in India in medical anthropology and beyond. The latter addresses psychiatry as originally a colonial and now a neocolonial practice, ignoring local contexts and overly relying on pharmaceuticals and ECT. They also stress mental health pluralism as resource and Ayurveda and ritual healing practices as more comprehensive and inclusive and less reductionist (Halliburton, 2009; Lang, 2018; Sax & Lang, 2021; Sébastia, 2009). The curious and experimenting psychiatrists we have described in this paper become allies in this critique. They too were not homogenous. While Dr John was more bold and willing to go ahead with experimentation, Dr Menon only imagined leaving the hospital and to start his own clinic though he did also encourage students training in ayurvedic approaches, and Dr Achuthan expressed respect for Ayurveda in mental health care. Although the curious intellectual and practical experimentation we described may have been influenced by the dynamics of the encounters with the anthropologists, they point towards dissatisfaction with biopsychiatric practice and their underlying assumptions in India amongst psychiatrists themselves. Their non-conforming or even counter-hegemonic ways of thinking and intervening in mental health also reflect the position of the World Psychiatric Association which aims to integrate religion and spirituality into psychiatric training and clinical practice (Moreira-Almeida et al., 2016), although the three psychiatrists we have described focus more on indigenous philosophies and healing approaches than on “religion” or “spirituality”. Whether these three practitioners represent idiosyncratic individuals going in their own directions in their practices, informed by local healing systems, or a more systematic or structural response to gaps in biomedical perspectives and practices remains to be examined. This would be a ripe area for exploration in future research that attends to the creative, experimental or non-conformist practices of psychiatrists.

Rather than simply applying a hegemonic biomedical psychiatry, these psychiatrists offer the possibility of a more locally-attuned, context sensitive psychiatric practice. Looking at diversity and experimentation, rather than conformity, reveals heterogenous, non-conforming, even counter-hegemonic practices and ideas. These reflect critique of decontextualization, ignorance of indigenous practices and pharmaceutical reductionism in medical anthropology and cross-cultural psychiatry. This perspective also provides tentative directions of where a de-colonial and future-oriented psychiatry might turn. These three practitioners give hope for more experimental, more inclusive, and contextually sensitivepsychiatric practices.

## Footnotes

1 The International Society for Psychological and Social Approaches to Psychosis (ISPS) is an international network of clinicians and academics who are critical of the biomedical model of psychosis and their journal *Psychosis* features a variety of critiques of this model by mental health professionals.

2 It should be added that Jadhav, one of the authors of the two Jain and Jadhav articles discussed above, is a critical psychiatrist, academic and medical anthropologist who edits a major medical anthropology journal *Anthropology & Medicine.* His critical skills aim in different directions from those cited in this paragraph though he follows in the tradition of Fanon in many ways and considers the psychological fallout of social hierarchies such as caste in India and class in the UK.

3 “Traditional Chinese medicine” has been described as in fact a variety of practices lumped into this rubric by biomedicine and outside observers (Zhan, 2009).

4 Some psychiatrists in China seem to prescribe traditional Chinese medicine for psychopharmaceuticals’ damaging effects, as anthropologist Zhiying Ma said in an interview in 2019, https://www.madinamerica.com/2019/09/recuperating-social-person-china-interview-zhiying-ma/.

5 Psychologists, too, go outside the (western) psychological model and some engage with South Asian disciplines of the mind. However we cannot go into this tangent in this paper.

6 See Halliburton (2009) and Lang (2018) for analyses of ayurvedic medical practices for treatment of psychopathology in Kerala.

## References

[CR1] Affleck W, Thamotharampillai U, Hinton D (2022). Walking Corpse Syndrome: A trauma-related idiom of distress amongst Sri Lankan Tamils. Transcultural Psychiatry.

[CR2] Baum E (2022). Embodied Minds: Hearts and Brains in Psychiatry and Chinese Medicine. Integrative Psychological and Behavioral Science.

[CR3] Biehl, J. (2013[2005]). *Vita: Life in a zone of social abandonment*. University of California Press.

[CR4] Breggin P (1991). Toxic psychiatry: Why therapy, empathy and love must replace the drugs, electroshock, and biochemical theories of the “new psychiatry”.

[CR5] Brodwin P (2013). Everyday Ethics: Voices from the Front Line of Community Psychiatry.

[CR6] Durkheim, E. (2014[orig. 1893]). *The division of labor in society* (H. D. Halls, Trans.). Free Press.

[CR7] Fanon, F. (2008 [orig. 1952]). *Black skin, white masks* (Trans.). Grove Press.

[CR8] Fernando, S. (2019 [orig. 1991]). *Mental health, race and culture*. Palgrave Macmillan.

[CR9] Halliburton M (2009). Mudpacks and Prozac: Experiencing ayurvedic, biomedical and religious healing.

[CR10] Jain S, Jadhav S (2008). A Cultural Critique of Community Psychiatry in India. International Journal of Health Services.

[CR11] Jain S, Jadhav S (2009). Pills that Swallow Policy: Clinical Ethnography of a Community Mental Health Program in Northern India. Transcultural Psychiatry.

[CR12] Jordan B, Davis-Floyd R (1993). Birth in Four Cultures.

[CR13] Lang C (2018). Depression in Kerala: Ayurveda and Mental Health Care in 21st Century India.

[CR14] Lang, C. (2019). Inspecting mental health. Depression, surveillance and care in Kerala, South India. *Culture, Medicine and Psychiatry, 43*(4), 596–612. 10.1007/s11013-019-09656-3 31729687

[CR15] Lee S (1999). Diagnosis Postponed: Shenjing Shuairuo and the Transformation of Psychiatry in Post-Mao China. Culture, Medicine and Psychiatry.

[CR16] Lock M, Nguyen V-K (2010). An Anthropology of Biomedicine.

[CR17] Mills C (2014). Decolonizing Global Mental Health: The Psychiatrization of the Majority World.

[CR18] Moncrieff J (2008). The Myth of the Chemical Cure: A Critique of Psychiatric Drug Treatment.

[CR19] Moreira-Almeida (2016). WPA Position Statement on Spirituality and Religion in Psychiatry. World Psychiatry.

[CR20] Naraindas H (2006). Of spineless babies and folic acid: Evidence and efficacy in biomedicine and ayurvedic medicine. Social Science & Medicine.

[CR21] Naraindas, H., Quack, J., & Sax, W. (Eds.). (2014). *Asymmetrical conversations: Contestations, circumventions, and the blurring of therapeutic boundaries*. Berghahn Books.

[CR22] Nunley M (1996). Why Psychiatrists in India Prescribe So Many Drugs. Culture, Medicine and Psychiatry.

[CR23] Pinto S (2014). Daughters of Parvati. Women and madness in contemporary India.

[CR24] Pinto S (2019). The doctor and Mrs. A.: Ethics and counter-ethics in an Indian dream analysis.

[CR25] Robcis C (2021). Disalienation: Politics, Philosophy, and Radical Psychiatry in Postwar France.

[CR27] Sax W, Lang C (2021). The movement for global mental health: Critical views from South and Southeast Asia.

[CR28] Sébastia B (2009). Restoring mental health in India: Pluralistic therapies and concepts.

[CR29] Scheid V (2013). Depression, Constraint, and the Liver: (Dis)assembling the Treatment of Emotion-Related Disorders in Chinese Medicine. Culture, Medicine and Psychiatry.

[CR30] Street A (2014). Biomedicine in an unstable place: Infrastructure and personhood in a Papua New Guinean Hospital.

[CR31] Szasz T (1961). The myth of mental illness: Foundations of a theory of personal conduct.

[CR32] Varma S (2020). The occupied clinic. Militarism and care in Kashmir.

[CR33] Zhan M (2009). Other-Worldly: Making Chinese Medicine through Transnational Frames.

[CR34] Zhang Li (2020). Anxious China: Inner Revolution and Politics of Psychotherapy.

